# The impact of a speaker’s voice quality on auditory perception and cognition: a behavioral and subjective approach

**DOI:** 10.3389/fpsyg.2023.1243249

**Published:** 2023-11-30

**Authors:** Isabel S. Schiller, Lukas Aspöck, Sabine J. Schlittmeier

**Affiliations:** ^1^Work and Engineering Psychology, Institute of Psychology, RWTH Aachen University, Aachen, Germany; ^2^Institute for Hearing Technology and Acoustics, RWTH Aachen University, Aachen, Germany

**Keywords:** Heard Text Recall, auditory Verbal Serial Recall, listening effort, listening comprehension, voice quality, hoarseness, voice perception, speech in noise

## Abstract

**Introduction:**

Our voice is key for conveying information and knowledge to others during verbal communication. However, those who heavily depend on their voice, such as teachers and university professors, often develop voice problems, signaled by hoarseness. The aim of this study was to investigate the effect of hoarseness on listeners’ memory for auditory-verbal information, listening effort, and listening impression.

**Methods:**

Forty-eight normally hearing adults performed two memory tasks that were auditorily presented in varied voice quality (typical vs. hoarse). The tasks were Heard Text Recall, as part of a dual-task paradigm, and auditory Verbal Serial Recall (aVSR). Participants also completed a listening impression questionnaire for both voice qualities. Behavioral measures of memory for auditory-verbal information and listening effort were performance and response time. Subjective measures of listening effort and other aspects of listening impression were questionnaire rating scores.

**Results:**

Results showed that, except for the aVSR, behavioral outcomes did not vary with the speaker’s voice quality. Regarding the aVSR, we found a significant interaction between voice quality and trial, indicating that participants’ recall performance dropped in the beginning of the task in the hoarse-voice condition but not in the typical-voice condition, and then increased again toward the end. Results from the listening impression questionnaire showed that listening to the hoarse voice resulted in significantly increased perceived listening effort, greater annoyance and poorer self-reported performance.

**Discussion:**

These findings suggest that hoarseness can, at least subjectively, compromise effective listening. Vocal health may be particularly important in the educational context, where listening and learning are closely linked.

## 1 Introduction

The phenomenon that speakers automatically raise their voice and adapt their speaking style in noisy environments is known as the Lombard effect (Lombard, 1911; Garnier and Henrich, 2014; Bottalico et al., 2017). Lombard speech may temporarily improve speech-in-noise intelligibility, but frequent vocal overuse can eventually lead to voice disorders (Byeon, 2019). Voice disorders often concern professional voice users, such as teachers and university professors, who heavily rely on their voice during work (Moghtader et al., 2020). A recent meta-analysis estimated the prevalence of voice disorders among university professors at 41% (Azari et al., 2022) compared to only about 6% among the general population (Roy et al., 2004). The main perceptual symptom of a voice disorder is an impaired voice quality (dysphonia), commonly referred to as hoarseness. Hoarseness may not only have negative consequences for those concerned, but also for others communicating with them. This study investigates the effect of hoarseness on memory for auditory-verbal information, listening effort, and subjective listening impression among adult listeners.

While background noise is widely acknowledged as a major obstacle to effective listening, an acoustically impaired speech signal, such as hoarseness, can pose significant barriers as well. Listening to an impaired voice compared to a typical (modal) voice can reduce speech intelligibility (Evitts et al., 2016; Ishikawa et al., 2017, 2018, 2021; Porcaro et al., 2020; Bottalico et al., 2021), consume cognitive resources (Imhof et al., 2014), slow down listeners’ processing speed (Evitts et al., 2016; Bottalico et al., 2021), and impair their memory for heard content (Imhof et al., 2014). Listeners have also shown more negative attitudes toward speakers with voice impairments (Amir and Levine-Yundof, 2013; Imhof et al., 2014). In the following, we will outline these consequences in more detail, focusing particularly on higher education.

When students listen to their professor, an essential prerequisite for learning is being able to understand the speech signal from an auditory-perceptual perspective (speech intelligibility) but also to comprehend and remember heard text. To date, relatively little is known regarding the effect of hoarseness on adult listeners’ memory for heard text. Imhof et al. (2014) studied university students’ memory recall for content information of stories that were either presented in a typical voice or a creaky voice. A creaky voice is characterized by a low and rattling vocal quality also known as vocal fry or pulse phonation (Anderson et al., 2014), while hoarseness rather refers to a rough and breathy voice quality (Garrett and Ossoff, 1995). The authors found that students remembered fewer content information when they listened to the creaky voice than the typical voice. In another study, Evitts et al. (2016) assessed adult listeners’ performance on content-related yes/no questions after they had listened to stories presented either in typical voice or dysphonic voice, which was mainly rough and strained in quality. Contrary to Imhof et al. (2014), these authors found no significant effect of voice quality on listeners’ task performance, although processing times were longer when participants were exposed to the dysphonic voice. The present study expands on these findings. Considering that professors use their voice to convey knowledge to their students, the present study seeks to further explore whether, how, and when hoarseness can compromise effective listening.

It has been found that a talker’s impaired voice quality can impede speech intelligibility, especially in noisy settings (Ishikawa et al., 2017, 2018, 2021; Bottalico et al., 2021). Acoustically, hoarseness is characterized by a devoicing of voiced phonemes (Schoentgen, 2006) and increased noise components blurring the contrasts between phonemes (Ishikawa et al., 2017). This is particularly critical in terms of vowel intelligibility (Ishikawa et al., 2018, 2021), with low vowels (e.g., /ae/ in “bag,” /ε/ in “bed,” and /∧/ in “sun”) being even more disrupted by dysphonia than high vowels (e.g., /i/ in “see” or /u/ in “true”). These acoustic features of hoarseness may lead to perceptual ambiguities that listeners must resolve, which requires additional processing resources and the division of attentional resources. The increased listening effort necessary to process an impaired voice quality is therefore likely to impact on the comprehension and retention of auditory-verbal information as well.

Listening effort refers to the mental effort or cognitive resources necessary to achieve a listening task, such as processing speech. Prolonged effortful listening can eventually lead to mental fatigue – a state of increased cognitive exhaustion (McGarrigle et al., 2014). The degree of listening effort can vary depending on factors such as the clarity and quality of the speaker’s voice, the presence of background noise or distractions, the complexity of the speech content, the listener’s own cognitive abilities and prior knowledge, as discussed in the Ease of Language Understanding (ELU) model (Rönnberg et al., 2013) and the Framework for Understanding Effortful Listening (FUEL; Pichora-Fuller et al., 2016). Listening effort may be assessed with behavioral methods [e.g., accuracy or response time (RT) measures; Houben et al., 2013; Gagné et al., 2017], subjective methods (e.g., self-reports; Fraser et al., 2010), physiological measures (e.g., pupil dilation; Zhang et al., 2021), and neuroimaging techniques (e.g., MRI; Rosemann and Thiel, 2020). In this article, we focus on behavioral and subjective methods.

A typical paradigm used for behaviorally measuring listening effort is the dual-task paradigm (DTP; Imhof et al., 2014; Gagné et al., 2017; Fintor et al., 2022). DTPs inherit the idea that cognitive capacity is limited and may be deliberately allocated between tasks (Kahneman, 1973; Pichora-Fuller et al., 2016). As indicated by the name, two tasks are performed in parallel. The primary task is the listening task and the secondary task is often a visual task (e.g., judging numbers on a screen; Fintor et al., 2022). Typically, a decrease in performance or an increase in RT in the secondary task in a challenging listening condition, while performance in the primary task remains unaffected, is interpreted as an indicator for increased listening effort (e.g., Imhof et al., 2014; Gagné et al., 2017; Fintor et al., 2022).

So far, the only study that has assessed the effect of a speaker’s voice quality on listening effort in adults was conducted by Imhof et al. (2014). Listening effort was investigated with a DTP that combined a listening comprehension and memory task (primary task) and a pen-and-paper attention task (secondary task). In confirmation of the authors’ hypothesis, listeners performed significantly worse in the secondary task when the listening task was presented in a creaky voice compared to a typical voice. This finding was explained in light of the Cognitive Load Theory (CLT; Paas et al., 2003; Paas and Ayres, 2014). Imhof et al. (2014) assumed that processing the creaky voice had increased listeners’ processing demands in the primary (memory) task, thus, leaving fewer resources available to perform well in the secondary task.

Regarding subjective methods, there is currently no standardized tool for assessing listening effort. Typically, perceived listening effort, as arising from background noise or a degraded speech signal, has been assessed with rating scales (Fraser et al., 2010; McAuliffe et al., 2012; Imhof et al., 2014). For example, authors have engaged listeners with Visual Analog Scales (McAuliffe et al., 2012) or rating scales from 0% (*no effort*) to 100% (*very effortful*; Fraser et al., 2010) to assess the listening effort. In the study conducted by Imhof et al. (2014), participants rated the degree of “listenability” (a term used interchangeably with listening effort) of stories read aloud in either a typical or creaky voice, using a scale ranging from 0 (*not listenable at all*) to 5 (*completely listenable*). Subjectively rated listenability was found to be significantly lower in the creaky voice than the typical voice. Overall, subjective ratings offer the advantage of being direct, and easy to administer and interpret, thus, constituting a valuable complement to behavioral measures of listening effort.

The quality of a speaker’s voice shapes our perception of a listening situation and influences which attributes we associate with the speaker. Research has repeatedly shown that speakers with voice impairments are perceived more negatively than those with typical voices (Amir and Levine-Yundof, 2013; Imhof et al., 2014). In the study by Imhof et al. (2014), listeners judged a female speaker to be significantly less attractive, dynamic, interesting, extraverted, emotional, relaxed, healthy, in a state of well-being, and strong when she spoke with a creaky voice, as compared to her habitual voice. This is in line with a study by Amir and Levine-Yundof (2013) in which listeners estimated dysphonic speakers as, for example, less successful, smart, sociable, and decisive than vocally healthy speakers. Shifting the focus from speaker perception to listening impression, the present study explores the effect of voice quality on perceived (1) listening effort, (2) concentration, (3) noise annoyance, (4) voice annoyance, (5) fatigue, (6) noise-induced performance drops, (7) voice-induced performance drops, and (8) need for recovery.

The goal of this study was to investigate the influence of hoarseness on adult listeners’ memory for auditory-verbal information, listening effort, and subjective listening impression. We conducted a laboratory study in which participants performed two memory tasks, one that involved listening to and remembering content information from spoken text, and the other one being auditory Verbal Serial Recall (aVSR). The speaker’s voice quality was varied between typical and hoarse. Listening effort was assessed in a dual-task paradigm. Listening impression regarding each voice quality was assessed with a questionnaire. Three hypotheses were tested: (H1) recall performance in the memory tasks decreases under the hoarse voice quality; (H2) in the dual-task paradigm, secondary task performance decreases and/or RT increases under the hoarse voice quality compared to the typical voice quality, indicating increased listening effort; (H3) listening to a hoarse voice impedes subjective listening impression, as compared to the typical voice.

## 2 Materials and methods

The study was approved by the Ethics Committee of the Faculty of Arts and Humanities, RWTH Aachen University (ref.: 2021_013_FB7_RWTH Aachen). The experiment was computer-based, programmed in Psychopy v2021.2.3 and run on a Dell Latitude 3590 laptop. The total duration was about 1 h. Prior to the experiment, participants provided written informed consent.

### 2.1 Participants

According to an *a priori* power analysis with GPower (Faul et al., 2009), 36 participants were necessary for a power of 0.95 at an α-level of 0.05 with an estimated medium effect size of *f*^2^ = 0.25. We recruited 50 participants to accommodate potential dropouts and corrupt data. Two participants were excluded, because they did not follow the task instructions correctly. Data analysis was carried out on the remaining 48 participants (38 females, age *M* = 23 years, range = 18–40 years), all of whom complied with the inclusion criteria: (a) normal or corrected-to-normal vision and, (b) proficiency in German at a native speaker level or equivalent, and (c) normal hearing, verified by hearing thresholds of ≤20 dB HL at octave frequencies between 500 and 4,000 Hz, assessed in an audiometry screening (ear3.0 audiometer, Auritec). Participation was compensated with a small payment or study credits.

### 2.2 Tasks and stimuli

Memory for auditory-verbal information was determined with two tasks, Heard Text Recall (HTR; Ermert et al., 2023; Schlittmeier et al., 2023) and aVSR (e.g., Hughes et al., 2016; Schlittmeier et al., 2021), listening effort was measured with a DTP, including the HTR as a primary task, and listening impression was assessed with a questionnaire. These tasks are explained below. Moreover, we quantified participants’ individual noise sensitivity with the NoiSeQ-R (Schutte et al., 2007; Griefahn, 2008) to control for this variable in our statistical analysis. Individual noise sensitivity was assessed, because it can influence performance in cognitive tasks performed under acoustically challenging conditions (Belojević et al., 1992).

The HTR involved listening to several stories (*n* = 13, ∼1 min each) about different families, where details like names and degree of kinship between family members are embedded into a coherent storyline. After each text presentation, participants were asked to answer nine content questions in 1–2 words (for a detailed task description, see Schlittmeier et al., 2023). Each correct answer was coded as 1, each false answer as 0. The aVSR involved listening to random sequences of nine digits between 1 and 9 (*n* = 22, including two practice sequences) and orally repeating back the correct sequence after a 10 s retention interval. Each digit correctly recalled at their right sequence position was coded as 1, each false response as 0.

We employed both HTR (Ermert et al., 2023; Schlittmeier et al., 2023) and aVSR (e.g., Hughes et al., 2016; Schlittmeier et al., 2021) to evaluate listeners’ memory for auditory-verbal information, because both tasks tap into the cognitive processes necessary for comprehending spoken language, such as when listening to university lectures. The HTR involves word identification, semantic and syntactic processing, forming mental content representations, and potentially integrating them into existing knowledge. This is crucial for understanding running speech and extracting meaning from it. The aVSR represents a highly controlled task for assessing short-term memory and sequential processing, likewise important for learning auditorily presented (i.e., heard) information. These processes are important in the academic context, for example, allowing students to follow the verbally presented flow of information. Compared to the HTR, the aVSR is a well-established task (e.g., Surprenant, 1999; Parmentier et al., 2006; Schlittmeier et al., 2008), making it advantageous in terms of measurement reliability. The HTR, on the other hand, enables the evaluation of more complex cognitive processing and might hold greater ecological validity. It also resembles the listening task employed by Imhof et al. (2014). Both tasks complement each other and allow us to assess the effect of hoarseness on listener’s memory for auditory-verbal information in a more comprehensive manner.

Listening effort was assessed in a DTP which included the HTR as a primary task and number judgment as a secondary task. For the secondary task, digits between 1–4 and 6–9 were visually presented on a computer screen in random order. Participants were instructed to indicate, via keypress, whether the respective digit was smaller or larger than five. Each correct response was coded as 1, each false response as 0. In addition to this binary performance measure, we assessed participants’ response time in the secondary task (i.e., the elapsed time between digit presentation to key response). The maximum response time was 1.5 s, afterward, the missing of a response was recorded and coded as 0 and the next digit appeared on the screen. Both HTR and number judgment were presented in single-task baseline conditions and dual-task conditions.

Additionally, we assessed participants’ listening impression with respect to both voice qualities based on eight questionnaire items. These items were presented in German but are reported in English for the purpose of this article: (1) How strong was your listening effort?, (2) How difficult was it for you to stay focused?, (3) How much did you feel disturbed or annoyed by background noise?, (4) How much did you feel disturbed or annoyed by the speaker’s voice?, (5) How exhausted do you feel right now?, (6) Was your cognitive performance impeded by the background noise?, (7) Was your cognitive performance impeded by the speaker’s voice?, and (8) How in need of recovery do you feel right now? Participants rated each item using a 5-point Likert scale with alphanumerical and verbal labels which translated to: 1 = *not at all*; 2 = *slightly*; 3 = *moderately*; 4 = *very much*; 5 = *extremely*.

Speech stimuli for the listening tasks (HTR and aVSR) originated from a 34-year-old German female speaker, recorded in a hemi-anechoic chamber at the Institute of Hearing Technology and Acoustics (RWTH Aachen University), using a condenser microphone (DPA 4066-OC-A-F00-LH) with a digital audio interface (Hammerfall DSP Multiface II, RME). The speaker first recorded the speech stimuli in her habitual voice and then, while imitating a hoarse voice. Both voice qualities were later evaluated by five speech-language pathologists, specialized in voice disorders, in a perceptual rating using the Grade Roughness Breathiness Asthenia Strain [Instability] [GRBAS(I)] scale (Hirano, 1981; Dejonckere et al., 1996). In an additional acoustic analysis, we determined the Acoustic Voice Quality Index (Maryn et al., 2010). The AVQI was calculated based on one voice sample per voice quality, consisting of 8 s of continuous speech and 3 s of a sustained vowel. Results of the perceptual and acoustic voice quality evaluation are presented in Table 1. Taken together, both analyses confirmed that (a) the speaker’s habitual voice was unimpaired, and (b) her simulated hoarse voice was moderately impaired. The hoarse voice was particularly characterized by roughness, strain, and instability, which are acoustically linked to factors such as increased noise components in the spectrum, as well as pitch and amplitude fluctuations (see e.g., Schoentgen, 2006). All speech stimuli were presented at an RMS level of 65 dB SPL, scaled using the software Praat (Boersma and Weenink, 2023). A speech rate analysis of the HTR texts revealed that the speaker spoke slightly faster in her typical voice (*M* = 2.8 words/s, SD = 0.21) than in her hoarse voice (*M* = 2.6 words/s, SD = 0.13). According to a paired-sample *t*-test, this difference was significant, *t*(23) = 2.968, *p* < 0.01. However, as inter-sentence intervals were always set to 600 ms, we considered this difference as negligible.

**TABLE 1:** Perceptual and acoustic evaluation of the typical and hoarse voice quality.

Perceptual evaluation (*n* = 5 raters)		
GRBAS(I) scale^a^	Typical voice Mdn^c^ (range)	Hoarse voice Mdn (range)
G (grade)	0 (0–1)	2 (1–2)
R (roughness)	1 (0–1)	2 (N/A)
B (breathiness)	0 (N/A)	0 (0–2)
A (asthenia)	0 (0–1)	1 (1–3)
S (strain)	1 (0–2)	2 (1–3)
I (instability)	0 (0–1)	2 (1–3)
**Acoustic evaluation**		
	**Typical voice**	**Hoarse voice**
AVQI^b^	2.39	3.55

^a^The GRBAS(I) scale (Hirano, 1981; Dejonckere et al., 1996) is a 4-point rating scale for assessing voice quality, ranging from 0 (*typical voice quality*) to 3 (*severe dysphonia*). GRBAS(I) ratings were performed by five speech-language pathologists.

^b^The AVQI (Maryn et al., 2010) is a tool for objective voice quality quantification, based on acoustic characteristics such as pitch, jitter, shimmer, and harmonics-to-noise ratio. AVQI scores range from 0 to 10, with lower sores indicating a better voice quality and higher values indicating a poorer voice quality. The cut-off value in German for distinguishing between typical and dysphonic voice quality is 3.05 (Maryn et al., 2014).

^c^Mdn, median.

Both HTR and aVSR were presented via headphones (Sony WH-1000XM3). To simulate similar listening conditions as during a university lecture, speech stimuli were merged with realistic background noise, leading to an SNR of ∼13 dB, which can be interpreted as a low to medium noise disturbance. The background noise had been binaurally recorded by placing an artificial head (Schmitz, 1995) in an occupied seminar room at the Institute of Hearing Technology and Acoustics (RWTH Aachen University) and included ventilation noise, unintelligible speech, and other distracting sounds such as rustling paper, moving chairs, or typing on a keyboard. Target speech signals were binaurally rendered based on simulated impulse responses of the corresponding speaker and receiver positions in the virtual seminar room. The simulation was created using RAVEN software (Schröder and Vorländer, 2011), which simulated the acoustic impression as if the speaker would speak to the listeners from a front position in the very same seminar room. The reverberation time of the simulated room was adjusted to the measured reverberation time (T30 = 0.7 s) using an interactive procedure (Aspöck, 2020). The presentation level for the experiment was set to 65 dB (A), calibrated with an artificial head from the Institute for Hearing Technology and Acoustics.

### 2.3 Procedure

Participants were individually tested in a soundproof booth (Studiobox premium) at the teaching and research area of Work and Engineering Psychology, RWTH Aachen University. Prior to the main experiment, an audiometry screening ensured that all participants had normal hearing. For the main experiment, participants were seated in front of a computer screen, connected to a keyboard that was placed on the table. The experiment consisted of two blocks, separated by a short break, each containing the HTR, aVSR, and listening impression questionnaire. Participants were told that, in each block, presented in different voice qualities, they would perform two listening tasks in the presence of moderate background noise. Counterbalanced across participants, one block was presented in a typical voice quality, the other one in a hoarse voice quality. Regarding the HTR, texts were balanced across blocks to ensure that no participant listened to the same text more than once.

Participants were instructed that, in the HTR task, they would listen to several stories about families, detailing the relationships, leisure activities, and professions of their members. After each story, nine content questions would appear on the screen and they should type in a short answer of 1–2 words using the keyboard. No time delay was included after the end of a story and before the first question was presented. Participants were informed that this task would be presented alone, and together with a number judgment task. Regarding number judgment, digits between 1–4 and 6–9 would appear on the screen and participants would be asked to indicate, via keypress, whether the digit was smaller or larger than five. For the aVSR, participants were told they would hear sequences of nine random digits between 1 and 9, each followed by a short retention interval. Their task would be to remember the digits in their correct order and orally repeat them after the retention interval. We told the participants that they would be asked to evaluate their listening impression at four occasions, (1) after the HTR and (2) the aVSR in the first block, and (3) after the HTR and (4) the aVSR in the second block. Their final task would be to complete the noise sensitivity questionnaire.

### 2.4 Statistical analysis

Data analysis was conducted using R (R Core Team, 2023). The key response variables related to the two voice qualities, typical and hoarse, were performance in the HTR task (binary variable: 1 for correct answers and 0 for incorrect answers), performance in the secondary task (binary variable: 1 for correct number judgments and 0 for incorrect judgments), response times of correct trials in the secondary task (measured in milliseconds from number presentation to keypress), performance in the aVSR (binary variable: 1 for correctly recalled digits and 0 for false responses), and rating scores from the listening impression questionnaire.

Performance and RT data were modeled with generalized linear mixed-effect models (GLMMs), using the *lme4* package (Bates et al., 2015). We chose this approach over traditional ANOVAs because GLMMs are more flexible, statistically powerful, and better capture individual-level variability and dependencies between observations (Jaeger, 2008; Lo and Andrews, 2015). GLMMs do not require data transformation to yield a normal distribution, and are therefore suitable for the analysis of binary and RT data, the latter of which is usually positively skewed (Whelan, 2008). Prior to RT data analysis, we identified and removed outliers that exceeded two standard deviations from the mean, following the procedure outlined in Berger and Kiefer (2021). During this process, we excluded 5.7% of the RT data. For the GLMMs modeling performance, we specified binomial distributions and logit link functions, while RT was modeled with a Gamma distribution and log link function.

Four different GLMMs were built to model the effect of *voice quality* on performance in the HTR, performance and RT in the secondary (number judgment) task, and performance in the aVSR. For the GLMM modeling HTR performance, we considered *voice quality* (typical vs. hoarse), *task condition* (single-tasking vs. dual-tasking), *trial* (referring to each subsequent text in a block), and all two-way and three-way interactions as fixed factors, and *participant ID*, individual *NoiSeQ-R score*, *item* (question), and *item* nested within *text* as random (intercept) factors. For the GLMM modeling secondary task performance and RT, we considered *task condition* [single-tasking (i.e., judging numbers without HTR in parallel) vs. dual-tasking with HTR presented in typical voice vs. dual-tasking with HTR presented in hoarse voice], *trial*, and their interaction as fixed factors, and *participant ID* and *NoiSeQ-R score* as random (intercept) factors. Finally, for the GLMM modeling aVSR performance, we considered *voice quality, trial* (referring to each subsequent sequence in a block), and their interaction as fixed factors, and *participant ID, NoiSeQ-R score*, and *position* (referring to the position of a digit in a respective sequence) nested within *trial* as random (intercept) factors. We identified the final GLMMs through forward model selection, comparing the different models with likelihood ratio tests.

We detailed our examination of GLMM assumptions. Specifically, residual plots revealed no apparent patterns against the fitted values, indicating that the assumption of constant variance (homoscedasticity) was met. Only regarding the GLMM modeling RT in the secondary (number judgment) task, the scatter plot showed some degree of heteroscedasticity and deviation from a normal distribution, probably because RT data was positively skewed. However, this deviation was not extreme. Whenever applicable, we conducted *post hoc* pairwise comparisons based on estimated marginal means (emmeans) using the *emmeans* package (Lenth, 2023). Regarding the subjective data, the effect of *voice quality* (typical vs. hoarse) on each of the eight items in the listening impression questionnaire was calculated with non-parametric Wilcoxon signed-rank tests, considering that these data were not normally distributed. Effect sizes were estimated with Cohen’s *d*.

## 3 Results

### 3.1 Effects of voice quality on memory for auditory-verbal information

The effect of *voice quality* on memory for auditory-verbal information was investigated based on performance in the HTR and aVSR. A descriptive analysis of participants’ performance in the HTR revealed that participants answered 58.2% (SD = 19.1%) of the questions correctly when listening to the typical voice and 56.5% (SD = 20.6%) when listening to the hoarse voice. With respect to *task condition*, participants’ percentage of correct answers was 62.8% (SD = 20.7%) during single-tasking for the normal-voice condition compared to 57.5% (SD = 23.4%) for the hoarse-voice condition, and 53.6% (SD = 16.3%) during dual-tasking for the normal-voice condition compared to 55.5% (SD = 17.7%) for the hoarse-voice condition. Table 2 provides a summary of the final GLMM that modeled HTR performance. The best-fitting model included *voice quality* and *task condition* as fixed effects and *participant ID* and *item* as random intercepts. Contrary to our hypothesis, there was no significant effect of *voice quality* on performance [χ^2^(1) = 0.01, *p* = 0.93]. However, we did find a significant effect of *task condition* [single- vs. dual-tasking; χ^2^(1) = 8.05, *p* = 0.004], which we further assessed in a *post hoc* analysis using *emmeans* package (Lenth, 2023). This pairwise comparison indicated that, irrespective of *voice quality*, performance was significantly better during single-tasking compared to dual-tasking (*z*-ratio = 2.84, *p* = 0.004).

**TABLE 2:** Results from the final GLMM modeling performance in the HTR task as predicted by *voice quality*.

	Fixed effects				
	Estimate	SE	*z*	95% CI	*p*
Intercept	0.48	0.18	2.72	0.14, 0.83	0.007**
**Voice quality**					
Typical	Reference				
Hoarse	−0.01	0.07	−0.09	−0.15, 0.13	0.93
**Task condition**					
ST^a^ baseline	Reference				
DT^b^	−0.25	0.09	−2.83	−0.43, −0.08	0.005**
	**Random effects**				
	**Variance**			**SD**	
Participant (intercept)	0.52			0.72	
Item (intercept)	1.17			1.08	

Number of observations: 4,320, groups: item = 90; participant = 48.

*p*-Values for fixed effects calculated using parametric bootstrapping. **p < 0.01.

Confidence intervals calculated using the Wald method. Model equation: performance ∼ voice quality + task condition + (1| participant) + (1| item); family = binomial, link function = logit.

^a^ST, single task.

^b^DT, dual task.

**FIGURE 1:**
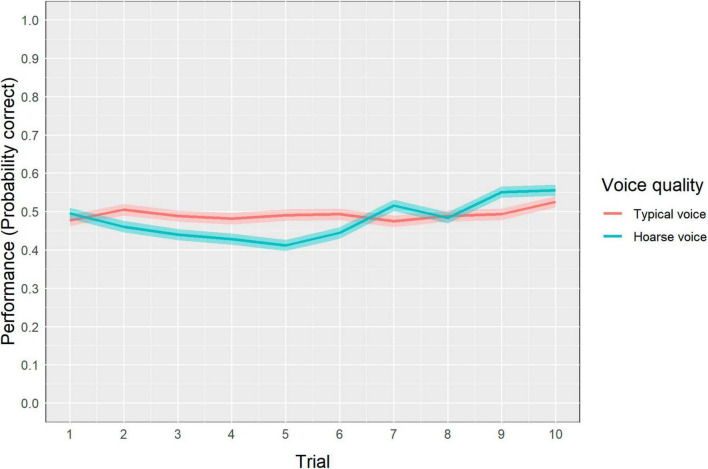
Interaction between *voice quality* and *trial* in the aVSR task. This graph shows participants’ recall performance across all trials in each voice-quality block respectively. Shaded areas refer to the 95% CIs.

Regarding the aVSR, a descriptive analysis indicated that the mean number of correctly recalled digits in a sequence was 4.4 out of 9 (SD = 1.5) in the typical-voice condition, compared to 4.3 out of 9 (SD = 1.3) in the hoarse-voice condition. The GLMM results for aVSR performance are presented in Table 3. The best fitting model included *voice quality* as a fixed factor and *participant ID* and *NoiSeQ-R score* as random intercepts. Although the GLMM output shows a main effect of *voice quality*, this effect is no longer significant when accounting for the interaction between *voice quality* and *trial*, as indicated by a type II ANOVA of the model [χ^2^(1) = 6.42, *p* = 0.01]. This interaction is depicted in Figure 1, showing that, in the typical-voice condition, performance remained more or less stable across the duration of the task (i.e., 10 trials). However, in the hoarse-voice condition, performance exhibits an initial decline over the first five trials, followed by a relatively gradual increase over the remaining trials, ultimately reaching notably higher level at the end of the task compared to the beginning.

**TABLE 3:** Results from the final GLMM modeling performance in the aVSR task as predicted by *voice quality* × *trial.*

	Fixed effects				
	Estimate	SE	*z*	95% CI	*p*
Intercept	−0.37	0.26	−1.47	−0.57, 0.44	0.142
**Voice quality**					
Typical	Reference				
Hoarse	−0.31	0.11	−2.89	−0.52, −0.10	0.004**
**Trial**	0.05	0.04	1.41	−0.06, 0.08	0.159
**Voice quality: trial**					
Typical voice	Reference				
Hoarse voice	0.04	0.02	2.53	0.01, 0.08	0.011*
	**Random effects**				
	**Variance**			**SD**	
Participant (intercept)	0.52			0.72	
Trial:position (intercept)	0.94			0.97	

Number of observations: 8,640, groups: participant = 48, trial:position = 90.

*p*-Values for fixed effects calculated using Laplace approximations. *p < 0.05, **p < 0.01.

Confidence intervals calculated using the Wald method. Model equation: performance ∼ voice quality × trial + (1| participant) + (1| trial:position), family = binomial, link function = logit.

### 3.2 Effect of voice quality on objective measures of listening effort

**FIGURE 2:**
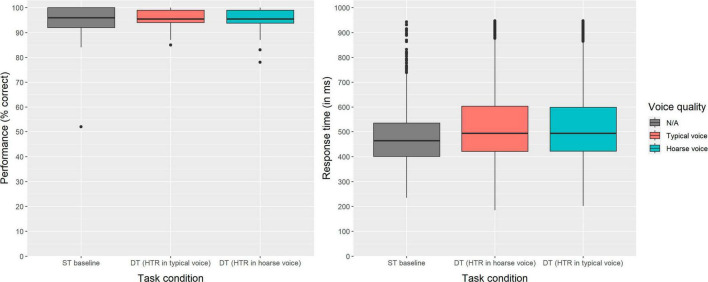
Effect of voice quality on performance **(left)** and response time **(right)** in the secondary task (number judgment). The lines inside the boxes represent the medians and the boxes represent the inter-quartile ranges (*IQR*). The whiskers extend to the minimum and maximum values within 1.5 times the *IQR*.

Behaviorally, the effect of *voice quality* on listening effort was assessed based on participants’ performance and response time in the secondary (number judgment) task. It was assumed that participants would perform significantly worse and take more time during dual-tasking, especially when the primary (HTR) task was presented in the hoarse voice compared to the typical voice. Figure 2 shows the descriptive results regarding performance (left) and RT (right) as a function of *task condition* (i.e., single-task baseline vs. dual-task with HTR in typical voice vs. dual-task with HTR in hoarse voice). As evident from this figure, performance was close to ceiling in all three conditions. The GLMM results for both outcome variables are provided in Table 4 (performance in the secondary task) and Table 5 (RT in the secondary task). Statistically, there was no significant effect of *task condition* on secondary task performance [χ^2^(2) = 1.221, *p* = 0.542], but a significant effect of *task condition* on RT [χ^2^(2) = 203.44, *p* < 0.001]. A *post hoc* analysis using *emmeans* package revealed that response times were significantly longer in both dual-task conditions compared to the single-task condition (*p*-values < 0.001), indicating that participants experienced an increased listening effort when performing the secondary task in parallel with the HTR. Importantly however, and in contrast to our hypothesis, the voice quality in which the HTR was presented did not make a difference (*z* = 1.91, *p* = 0.137).

**TABLE 4:** Results from the final GLMM modeling performance in the secondary (number judgment) task as predicted by *task condition*.

Fixed effects					
	Estimate	SE	*z*	95% CI	*p*
Intercept	3.22	0.16	20.05	2.89, 3.55	<0.001***
**Task condition**					
DT^a^ typical voice	Reference				
DT^b^ hoarse voice	−0.09	0.10	−0.90	−0.30, 0.11	0.37
ST Baseline	0.06	0.17	0.33	−0.28, 0.42	0.74
**Random effects**					
	**Variance**			**SD**	
Participant (intercept)	0.47			0.69	
NoiSeQ-R score (intercept)	0.13			0.37	

Number of observations: 9,549, groups: participant = 48; NoiSeQ-R score = 22.

*p*-Values for fixed effects calculated using Laplace approximations. ***p < 0.001.

Confidence intervals calculated using the Wald method. Model equation: performance ∼ task condition + (1| participant) + (1| NoiSeQ-R score), family = binomial, link function = logit.

^a^ST, single task.

^b^DT, dual task.

**TABLE 5:** Results from the final GLMM modeling response time in the secondary (number judgment) task as predicted by *task condition*.

Fixed effects					
	Estimate	SE	*z*	95% CI	*p*
Intercept	6.27	0.02	341.4	6.23, 6.30	<0.001***
**Task condition**					
DT^a^ typical voice	Reference				
DT^b^ hoarse voice	0.01	0.01	1.91	−0.00, 0.02	0.058
ST baseline	−0.10	0.01	−12.59	−0.11, −0.08	<0.001***
	**Random effects**				
	**Variance**			**SD**	
Participant (intercept)	0.02			0.12	

Number of observations: 8,582, groups: participant = 48. p-Values for fixed effects calculated using parametric bootstrapping. ***p < 0.001. Confidence intervals calculated using the Wald method. Model equation: response time ∼ task condition + (1| participant), family = Gamma, link function = log.

^a^ST, single task.

^b^DT, dual task.

### 3.3 Effect of voice quality on the subjective listening impression

**FIGURE 3:**
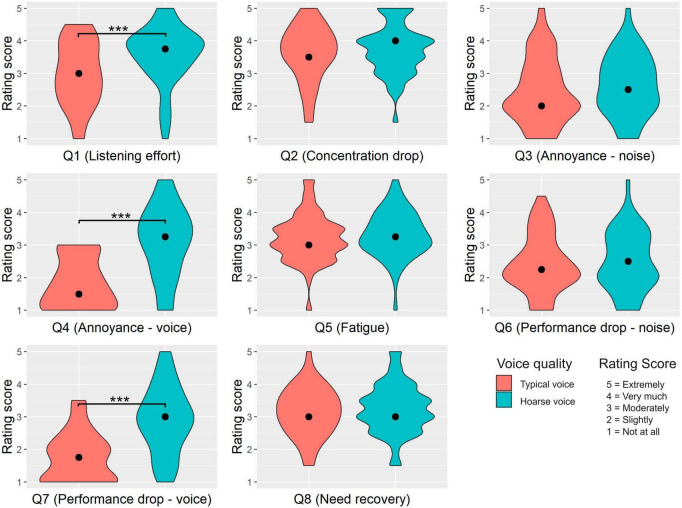
Effect of voice quality on subjective performance and listening impression. This figure shows violin plots of the data distributions from each question (Q) of the listening impression questionnaire (*N* = 48 participants). Rating scores are plotted against both voice qualities. The black dots refer to the medians. Significant differences are indicated by asterisks (***p < 0.001). The English translations of the questions were: (Q1) How strong was your listening effort?, (Q2) How difficult was it for you to stay focused?, (Q3) How much did you feel disturbed or annoyed by background noise?, (Q4) How much did you feel disturbed or annoyed by the speaker’s voice?, (Q5) How exhausted do you feel right now?, (Q6) Was your cognitive performance impeded by the background noise?, (Q7) Was your cognitive performance impeded by the speaker’s voice?, and (Q8) How in need of recovery do you feel right now?

To assess the effect of the speaker’s voice quality on subjective listening impression, participants responded to eight questions targeting listening effort, annoyance, fatigue, performance, attention, and speech-in-noise perception. The rating score distributions for each question as a function of *voice quality* are shown in Figure 3. Descriptive and statistical results of the comparisons between the typical and hoarse voice quality on the rating scores of each questionnaire item are presented in Table 6. As shown by the results of Wilcoxon’s signed rank test, we found a significant effect of *voice quality* for three questions. More precisely, the hoarse voice was perceived to be significantly more effortful to listen to (Question 1), more annoying than the typical voice quality (Question 4), and more impeding for cognitive performance (Question 7).

**TABLE 6:** Descriptive and inferential statistics for the rating scores in the listening impression questionnaire as a function of voice quality (*n* = 48).

Question	Typical voice		Hoarse voice		Wilcoxon’s signed rank test results	Cohen’s *d*
	Mdn^a^	IQR^b^	Mdn	IQR		
Q1: listening effort	3.0	1.62	3.75	1	*V* = 142, *p* < 0.001***	0.62
Q2: concentration drop	3.5	1	4	0.63	*V* = 214, *p* = 0.23	0.18
Q3: annoyance – noise	2	1	2.5	1.5	*V* = 250, *p* = 0.18	0.17
Q4: annoyance – voice	1.5	1.5	3.25	1.62	*V* = 9, *p* < 0.001***	1.34
Q5: fatigue	3	0.63	3.25	1	*V* = 250, *p* = 0.27	0.14
Q6: performance drop – noise	2.25	1	2.5	1.12	*V* = 195, *p* = 0.63	0.07
Q7: performance drop – voice	1.75	1	3	1.12	*V* = 8, *p* < 0.001***	1.24
Q8: need recovery	3	1	3	0.63	*V* = 210, *p* = 0.3	0.12

For all questions, a 5-point rating scale was given which ranged from 1 (*not at all*) to 5 (*extremely*). ***p < 0.001 (highly significant).

^a^Mdn, median.

^b^IQR, interquartile range.

## 4 Discussion

This study investigated the effect of a speaker’s voice quality on memory for auditory-verbal information, listening effort, and overall listening impression. Results showed that the speaker’s voice quality influenced listeners’ subjective perception. Exposure to hoarseness was linked to increased perceived listening effort, greater annoyance, and impeded cognitive performance. Despite our expectations, the behavioral outcomes did not vary with respect to voice quality, except for the significant interaction between voice quality and trial that was found regarding the aVSR. In the following, these results and their implications are discussed in more depth.

Contrary to our hypothesis, the speaker’s hoarse voice quality had no significant effect on listeners’ recall performance in the HTR task. This confirms the findings of Evitts et al. (2016), but contradicts those of Imhof et al. (2014). The latter study tested adult listeners’ memory for content information from stories and found that performance decreased under a speaker’s creaky voice. The discrepancy between our results and Imhof et al.’s (2014) findings could relate to the way the comprehension task questions were formulated and the resulting level of difficulty. Imhof et al. (2014) used multiple-choice questions, while the HTR (Ermert et al., 2023; Schlittmeier et al., 2023) uses open-ended questions. Multiple-choice questions are generally easier, because they require less active memory recall and prior knowledge (Ozuru et al., 2013; Polat, 2020). Indeed, almost half of the HTR questions in our study were answered incorrectly. In light of the Cognitive Load Theory (Paas et al., 2003; Paas and Ayres, 2014), one explanation could be that, in response to the challenging task and listening conditions, participants might have increased their efforts and invested additional cognitive resources to solve the listening task. Such a “reactive effort enhancement” (Kahneman, 1973) might have mitigated differing impacts of the two voice qualities in the HTR while promoting the differences in subjective ratings. It could also be that the perceptual difference between the typical and hoarse voice quality was not strong enough for the latter to seriously disturb the listeners. For our study, we chose a moderately hoarse voice over a severely dysphonic voice, aiming for a voice quality that could still be encountered in real-life teaching scenarios.

Regarding the aVSR, we found an intriguing interaction between voice quality and trial on recall performance. This interaction indicates that when exposed to the hoarse voice, participants’ performance initially decreased over the first half of the task, but then increased again over the second half, reaching a level even slightly above that in the beginning of the task. This was not true for the typical-voice condition for which recall performance remained relatively stable throughout the task. Perhaps, in the hoarse-voice condition, there was a novelty effect when participants first started the task and were exposed to what might have been perceived as an unusal voice quality. It could be that they were therefore initially more focused and motivated to perform well despite (or even because of) the challenging listening condition, enhancing their effort for a brief period of time (Kahneman, 1973). As they continued, the novelty effect might have faded, causing a dip in performance. Yet, as participants progressed further through the aVSR trials, they might have begun to devise strategies to better recall the digits while either coping with the hoarse voice or tuning out its “peculiarity.” This might explain the subsequent improvement in recall. Nevertheless, up to this point, this interpretation remains speculative, and further studies are necessary to delve deeper into the temporal effects of hoarseness on recall performance.

In terms of behavioral measures of listening effort, we assumed that the speaker’s hoarse voice quality would lead to poorer performance and/or longer response times in the secondary task of the DTP. However, unlike Imhof et al. (2014), we did not find such an effect. The fact that our results did reveal an increased *perceived* listening effort under the hoarse-voice condition suggests that we might have to re-evaluate the chosen DTP. If the primary task was too difficult, participants might have shifted their focus to perform well in the secondary task rather than expending more and more effort on the listening task. This notion is supported by a lower HTR performance during dual-tasking compared to single-tasking. On the other hand, literature proposes that when individuals become overwhelmed during the primary task, they tend to allocate their attention to the more manageable secondary task, even when instructed to prioritize the primary task (Zekveld et al., 2014; Wu et al., 2016). Generally, the aspect of task difficulty deserves more attention in future studies, as literature on child listeners suggests that the impact of impaired voice might diminish when the task becomes either too simple or excessively demanding (Lyberg-Åhlander et al., 2015). Apart from task difficulty, the FUEL (Pichora-Fuller et al., 2016) highlights the importance of motivation for effortful listening; maybe our participants’ motivation to engage in the difficult listening task was not high enough in this type of DTP. Another possible reason relates to the difference in speech rate between the typical and hoarse voice, with speech in the typical voice being about 7% faster. Due to this difference, participants had slightly more time for processing and retaining content information in the hoarse voice condition, so the potentially relieving effects of speech rate and impeding effects of voice quality might have been confounded. In addition to what we already discussed, it is worth noting that listening effort can also vary with listeners’ age (Kwak and Han, 2021). However, it is unlikely that this aspect explains the discrepancy between our findings and those of Imhof et al. (2014), as their participants had approximately the same age (mean = 24 years) as ours (mean = 23 years).

The listening impression questionnaire aimed to assess the impact of the speaker’s voice quality on individuals’ subjective experience during listening. Along with perceived listening effort, it included seven additional items. The results indicated that listeners experienced greater perceived listening effort, more annoyance and impeded cognitive performance when confronted with a hoarse voice compared to a typical voice. This finding relates to past studies which revealed that listeners have more negative attitudes toward dysphonic speakers in comparison to vocally healthy individuals (Amir and Levine-Yundof, 2013; Imhof et al., 2014). A hoarse speaker’s voice may trigger affective responses within listeners, influencing their willingness to engage in the listening task, similarly to what we previously discussed with regard to primary task difficulty. Notably, certain items within the listening impression questionnaire did not exhibit variations based on voice quality. These included participants’ ability to concentrate, noise-induced annoyance, perceived noise-induced performance decrements, fatigue and need for rest. The exact reasons behind this finding remain unclear. A follow-up study could investigate the effect of a speaker’s voice quality on listening impression using the same questionnaire but in a different context, for example, after longer listening tasks, such as an entire lecture, and maybe by integrating the visual modality.

### 4.1 Limitations

It is important to acknowledge several constraints of this study, which may have implications for the interpretation and application of the results. First, we conducted a purely auditory study in a highly controlled setting. In real life, perceiving, comprehending, and retaining auditory-verbal information is often an audio-visual process. Therefore, one step in pursuing this research thread will be to integrate the visual modality in future work. Second, the chosen listening tasks, aVSR and HTR, primarily evaluate short-term memory, even though many real-world listening scenarios, such as university lectures, also demand the long-term retention of the information heard. Nonetheless, we argue that short-term memory, specifically within the context of text comprehension as assessed by the HTR, serves as a fundamental basis for long-term storage and recall. The cognitive processes of encoding and initial retrieval, play a central role in the capacity to consolidate and retain information over an extended period. If a student struggles with crucial components like listening comprehension (as assessed by the HTR), sequential learning (as assessed by the aVSR), and immediate recall (requested by both aVSR and HTR), they are likely to encounter problems when long-term retention is required. In the future, it would be intriguing to incorporate an assessment of long-term recall into our research endeavors. Third, it is possible that the primary (listening) task in the DTP was too difficult, leading participants to optimize their performance in the secondary task. If this happened, secondary task results might not accurately indicate listening effort. In future studies that use the HTR as a primary task in DTPs, adding the visual cues of the speaker’s articulation may increase listeners’ motivation to engage and improve performance in this challenging listening task. The last limitation we wish to address is that acoustic and perceptual analyses of the typical and hoarse voice quality were not conducted on the entirety of the speech material participants encountered. As a consequence, we cannot provide information regarding potential voice quality variations across trials which might have influenced the behavioral outcomes.

## 5 Conclusion

This study aimed to investigate the effect of hoarseness on auditory-verbal working memory, listening effort, and subjective listening impression. Our findings suggest that a speaker’s hoarseness may subjectively disturb effective listening in terms of higher listening effort, more annoyance, and the impression that one’s performance suffers. This was not apparent in the behavioral measures of listening effort, which could have different reasons including that the disturbing effect of the hoarse voice was only subtle. An intriguing finding emerged from the interaction between voice quality and trial in verbal serial recall. It suggests that, in the typical-voice condition, performance did not change throughout the task, whereas in the hoarse-voice condition, it first dropped and then showed signs of recovery. Our observation raises the possibility that listeners might become used to a dysphonic voice over time, but this cautious speculation warrants further exploration. Overall, our findings have important implications in the context of university teaching and can inform future research on listening effort. University professors’ vocal health is crucial for effective teaching and requires ongoing monitoring for the benefit of both the professors and their students.

## Data availability statement

The raw data supporting the conclusions of this article will be made available by the authors, without undue reservation.

## Ethics statement

The studies involving humans were approved by the Ethics Committee of the Faculty of Arts and Humanities, RWTH Aachen University (2021_13_FB7_RWTH Aachen). The studies were conducted in accordance with the local legislation and institutional requirements. The participants provided their written informed consent to participate in this study.

## Author contributions

IS: conceptualization, methodology, data curation, investigation and supervision, formal analysis, writing – original draft, vizualization, and project administration. SS: methodology, writing – review and editing, and funding acquisition. LA: methodology and writing – review and editing. All authors contributed to the article and approved the submitted version.
